# Traumatic stress alters neural reactivity to visual stimulation

**DOI:** 10.1038/s44277-025-00030-3

**Published:** 2025-05-21

**Authors:** Nathaniel G. Harnett, Grace E. Rowland, E. Kate Webb, Tianyi Li, Soumyaa Joshi, Kerry J. Ressler, Isabelle M. Rosso

**Affiliations:** 1 Division of Depression and Anxiety, McLean Hospital, Belmont, MA USA; 2 Department of Psychiatry, Harvard Medical School, Boston, MA USA

**Keywords:** Risk factors, Cognitive neuroscience

## Abstract

Traumatic stress is a precursor to the development of posttraumatic stress disorder (PTSD). Emergent research suggests visual processing regions may be relevant to PTSD development; however, no previous research to date has investigated the potential effects of trauma exposure on neural reactivity to non-affective visual stimulation. In the present study, 24 recently trauma-exposed (RTE) and 16 without recent exposure to trauma (NRTE) individuals completed functional magnetic resonance imaging during alternating blocks of flickering checkerboard presentations and attention/rest with an attentional check. RTE participants were recruited within ~2–4 weeks of trauma, and PTSD symptoms were assessed both at the time of the magnetic resonance imaging scan and 6 months following trauma exposure. RTE participants showed greater deactivation within the visual cortex compared to NRTE participants. Further, NRTE participants showed greater neural reactivity within the dorsomedial prefrontal cortex during stimulation compared to attention/rest, while no difference was observed in RTE participants. Connectivity analyses also revealed that visual cortex to paracentral gyrus connectivity was greater during stimulation compared to attention/rest, but only for the NRTE participants. Finally, neural reactivity to visual stimulation was negatively associated with PTSD symptoms within the RTE group. Our findings suggest that trauma exposure is associated with acute alterations in the neural function that underlies basic visual processing. Furthermore, trauma-induced variability in visual circuit function may be related to the development and expression of PTSD symptoms.

## Lay Summary

This study investigated if exposure to traumatic stress altered brain activity to basic visual stimulation. The authors found that recent trauma survivors showed altered activity within the visual cortex compared to controls. Further, visual cortex reactivity was associated with PTSD symptoms. These findings suggest trauma exposure may disrupt more basic visual processing which may in turn contribute to PTSD symptom development.

## Introduction

Traumatic stress can result in cognitive and affective dysfunction in the form of posttraumatic stress disorder (PTSD). PTSD is typically thought to be associated with disrupted neurobiological function within core circuitry that supports threat-related processes such as the prefrontal cortex (PFC), hippocampus, and amygdala [1, 2]. However, emergent research suggests that sensory circuits – such as those that support visual processes – are also relevant to the disorder [3, 4]. Limited research to date, however, has probed the neural function of visual circuitry in the early aftermath of trauma to determine if responsivity within such circuitry is either associated with acute traumatic stress or predictive of PTSD symptom development. Further characterization of potential visual circuit dysfunction in recent trauma survivors may thus provide novel insight into the neurobiological mechanisms of PTSD susceptibility.

Prior PTSD research suggests that visual processing of stimuli may be disrupted and associated with neurobiological alterations within visual pathways. For example, previous work observed that visual stimuli (affective and neutral images) were associated with lower neural reactivity in those with PTSD compared to trauma-exposed controls within the visual cortex [5]. Conversely, separate studies observed higher neural reactivity to negative compared to neutral visual stimuli in those with PTSD [6, 7]. Further, PTSD is associated with altered coactivation patterns within both visual and threat processing networks. A recent report found that coactivation of the visual cortex and hippocampus in individuals with PTSD was associated with intrusion symptoms [8]. Similarly, connectivity between visual networks and prefrontal networks (e.g., frontoparietal network) at rest is predictive of PTSD symptom cluster scores [9]. Contrary findings in the current literature may be partially related to the types of stimulation used to assess affect-related circuitry and responses. Taken together, the prior literature suggests PTSD is associated with altered neural reactivity within both visual and threat processing networks in response to visual stimuli.

Limited work has investigated neural reactivity to visual stimuli in recent trauma survivors. The previous research largely focused on individuals with a longer chronicity of PTSD symptoms or time since trauma, which may not reflect associations in the acute phase following trauma. Findings from structural imaging studies suggest the transition from acute stress disorder to PTSD is partially mediated by reductions in gray matter volume of primary visual cortex [10]. Our prior work suggests that structural covariance of the ventral visual stream in recent trauma survivors is associated with PTSD symptoms [11, 12]. Similarly, we have observed that resting-state functional coupling between the visual cortex and amygdala/hippocampus in the first few weeks after trauma exposure is associated with 3-month PTSD symptoms when accounting for baseline symptoms [13]. Traumatic stress may therefore be associated with visual circuit neurobiology and development of PTSD symptoms. However, no research has specifically investigated neural reactivity to basic non-affective visual stimulation in recent trauma survivors and whether such reactivity may be related to PTSD symptom development. Prior work often examined reactivity to affective stimuli (e.g., negative or fearful images) compared with non-affective stimuli (e.g., neutral pictures or shapes), given PTSD symptoms are frequently tied to negative emotionality. However, it is also possible that the effects of traumatic stress and PTSD are not valence-specific, which may inform neurobiological conceptualizations of PTSD. Non-affective stimulation procedures, such as flickering checkerboards, are known to produce robust alterations within visual cortex and may be a useful probe for visual circuit function [14–16]. No research to date has explicitly utilized robust non-affective visual stimuli (e.g., flickering checkerboard) traditionally used to assess visual cortex function to measure potential impacts of recent trauma exposure on reactivity to neutral stimuli. Assessment of neural reactivity to basic, non-affective stimulation may therefore offer novel insight into the neurobiological mechanisms of PTSD susceptibility.

The present study investigated neural responsivity to visual stimulation as a function of recent trauma exposure and PTSD symptom severity. We measured the blood oxygen level-dependent (BOLD) response with functional magnetic resonance imaging (fMRI) during a flickering checkerboard paradigm in recent trauma survivors and individuals without recent trauma exposure. We also assessed participants’ self-reported PTSD symptoms at the time of the MRI session and 6 months later. We anticipated that trauma survivors would show altered BOLD responses in the visual cortex compared to control participants and that BOLD responses would correlate with PTSD symptoms. The current findings offer new insight into the acute effects of traumatic stress on the neurobiology of sensorial circuitry and the brain basis of PTSD susceptibility.

## Methods and materials

### Participants

Both recently trauma-exposed (RTE) participants and those without recent exposure to trauma (NRTE) participants were recruited for the present study. RTE participants were recruited from Emergency Departments (ED) within the Mass General Brigham hospital network via online research invitation or direct contact in participating EDs. Trauma was defined as a medical injury requiring admission to the ED. Details of broad class traumatic events experienced by participants are provided in the supplement (Table S1). NRTE participants were recruited via community posting. RTE participants were eligible provided they were admitted to a Mass General Brigham facility within the past 2–4 weeks for a traumatic event (M = 26.79 days, SD = 7.86 days). NRTE participants were included regardless of prior trauma history, provided the trauma had not occurred in the past 2–4 weeks. General inclusion criteria were: (a) participant aged 18–65, (b) ability to provide informed consent, (c) ability to read and speak English, (d) an ED Glasgow Coma Scale score of 15, and (e) normal or corrected-to-normal vision. Exclusion criteria were: prior intracranial bleeding or hemorrhage, current neurological disorder, mania, psychosis, current suicidal ideation, inability to abstain from drug or alcohol use for four hours prior to study visit, history of schizophrenia or schizoaffective disorder, blood disorder, or an MRI contraindication. Fifty participants were enrolled and consented for the study. Ten participants did not complete MRI and were not included in the present analysis. The final sample consisted of 40 participants (24 RTE, 16 NRTE; Table 1). All participants gave written informed consent as approved by the Mass General Brigham Institutional Review Board.

**Table 1:** Demographic information

	Recent trauma-exposed (RTE)	Non-recent trauma-exposed (NRTE)	χ2/T-statistic, (p-value)
*Demographics*	*n* = 24	*N* = 16	
Age	34.26 (14.67)	33.54 (14.69)	0.14, (0.892)
Sex assigned at birth			3.34, (0.068)
Female	11 (46%)	12 (75%)	
Male	13 (54%)	4 (25%)	
Race			0.73, (0.694)
White	13 (54%)	7 (43%)	
Black	5 (21%)	3 (19%)	
Other	6 (25%)	6 (38%)	
Highest grade level			0.37, (0.830)
At least high school education (graduate, GED, some college)	7 (29%)	4 (25%)	
At least college education (Occupational/Vocational/Associates/Bachelor’s degree)	10 (42%)	8 (50%)	
Graduate degree (Master’s, Professional, or Doctoral)	6 (25%)	3 (19%)	
Missing/Not Provided	1 (4%)	1 (6%)	
Income			0.62, (0.539)
Average	$119,489	$93,077	
Median	$68,000	$50,000	
*Clinical characteristics*			
PCL-5 scores			
2-week	25.74 (22.96)	7.53 (9.13)	2.91, (0.006)
6-month	13.06 (18.28)	6.29 (7.42)	1.30, (0.204)
PROMIS depression			
2-week	17.67 (8.44)	11.87 (5.07)	2.40, (0.021)
6-month	15.41 (7.85)	13.92 (6.98)	0.54, (0.594)
LEC-5 scores			
All Trauma	13.46 (7.96)	8.06 (7.22)	2.18, (0.036)
Past Trauma	12.46 (7.96)	8.06 (7.22)	1.77, (0.084)
CTQ-SF scores	41.95 (17.03)	33.69 (12.44)	1.65, (0.109)

χ^2^ test used for nominal data and independent t-test used for continuous data.

*CTQ-SF* childhood trauma questionnaire, *GED* general education development, *LEC-5* life events checklist for DSM-5, *PCL-5* PTSD checklist for DSM-5, *PROMIS* patient-reported outcomes measurement information system.

### Demographic and psychometric assessment

Participant demographics were assessed using a self-report form that indexed characteristics such as sex assigned at birth, race, ethnicity, age, income, and education. Participant race and ethnicity were assessed via self-report along several categories, with multiple selection allowed. Demographic data are reported in Table 1.

Participants reported lifetime trauma exposure using the Life Events Checklist for DSM-5 (LEC-5) [17] and the Childhood Trauma Questionnaire Short Form (CTQ-SF) [18]. The LEC-5 queries participants on exposure to potentially traumatic events that were (a) directly experienced, (b) witnessed by the participants, (c) learned about as happening to someone close to the participant, or (d) encountered through occupational exposure to details of the event due to occupation. Responses on the LEC-5 were summed to obtain an index of prior trauma load for each participant (Table 1). An index of “past trauma” was assessed for the RTE group by subtracting the current trauma from the total score. Traumatic event types experienced in each group are provided in the supplement (Table S2). The short-form version of the CTQ consists of 28 items to assess experiences of abuse (physical/emotional/sexual), neglect (physical/emotional), and minimization/denial. All items were rated on a Likert scale from 1–5, and the total score was calculated as the sum of the abuse and neglect subscales (accounting for reverse scored/negatively phrased items). PTSD symptoms were assessed using the PTSD Checklist for DSM-5 (PCL-5) [19]. The PCL-5 is a 20-item self-report questionnaire assessing symptom presence and severity. During the MRI visit, RTE participants were asked to answer questions related to the recent event that brought them to the ED, focusing on symptoms since the time of trauma. NRTE participants were asked to answer questions related to their worst traumatic event and symptoms over the past month. Both RTE and NRTE participants completed the PCL-5 and PROMIS measure again six months later to query symptoms experienced in the past month. Depression symptoms for the RTE and NRTE group were assessed using the Patient-Reported Outcomes Measurement Information System (PROMIS) Depression instrument from the PROMIS short form 8b [20] at the MRI visit and 6-month follow-up. Participants rated items on a Likert scale from 1–5, and the total score of items was used as the index of depression severity.

### Magnetic resonance imaging

Functional MRI data was collected using a multi-echo sequence (TR = 2000 ms, TEs = 13.00/30.22/47.44/64.66 ms, flip angle = 67 degrees, FOV = 208 mm, slices = 72, Voxel size = 2.4 mm × 2.4 mm × 2 mm) during visual stimulation (Fig. S1). The visual stimulation task consisted of alternating blocks of a flickering checkerboard and an attention/rest check. A full-field flickering checkerboard (8 Hz) with a yellow fixation cross was presented for 15 s. Immediately following, a red dot on a gray background was presented for 15 s. Participants were instructed to fixate on the yellow cross and to press a button on an MRI-compatible button box when the red dot was presented. Each block was presented 14 times. Preprocessing was completed using FMRIPREP version 21.1.1. Details of the preprocessing steps are provided in the supplement (Supplementary Material). For the present analyses, non-aggressively denoised outputs from AROMA were used for subsequent first-level models.

Primary first-level analyses to obtain participant-level maps of the BOLD response to stimuli were completed using the Analysis for Functional NeuroImages (AFNI; [21]). Preprocessed data were normalized such that amplitude deflections from the first-level models represented percent signal change. The first-level models included parameters for baseline drift, presentation of the checkerboard condition, presentation of the rest/red-dot condition, participant button presses, as well as global, white matter, and cerebrospinal fluid signal. The checkerboard and attention/rest conditions were modeled by convolving a boxcar function for the duration of the block with a canonical gamma-variate hemodynamic response function (HRF). Button presses were modeled by convolving the instantaneous regressor with the HRF. The other parameters were not convolved with an HRF.

### Statistical analyses

Statistical analyses were conducted using the JASP statistical software package [22] and AFNI [21]. Chi-square and independent samples t-tests were performed to evaluate between-group differences in demographic and psychometric assessments between the RTE and NRTE groups. Normality of psychometric assessments were assessed using a Shapiro-Wilks test (Supplementary Material). Voxelwise linear models were run using 3dMVM [23] to evaluate main effects of group (RTE versus NRTE), stimulus type (stimulation versus attention/rest), and a group-by-stimulus-type interaction on the BOLD response. Residuals from the first-level models were used to determine the spatial autocorrelation function (3dFWHMx) for each participant, and the average was used to define the spatial autocorrelation in 3dClustSim (10,000 iterations) and determine cluster extent (*k* = 172 needed to maintain a = 0.05 (cluster forming threshold of *p* = 0.005). The BOLD response was extracted for each participant within significant clusters from the voxelwise analysis and included in linear regression analyses to investigate the association between average or differential responses to stimuli with both acute and 6-month PTSD symptoms.

Two *ad hoc* exploratory analyses were performed to determine if neural connectivity may vary as a function of trauma exposure or stimulus type based on our findings from the above model. Using significant regions identified in the 3dMVM above, we first completed an analysis of background connectivity by correlating the timecourse of the residuals from the first-level models within the ROIs identified in the main analyses with the rest of the brain [24]. We also completed a general psychophysiological (gPPI) interaction analysis using ROIs identified in the primary analysis [25]. The gPPI involved detrending and deconvolving (with a standard HRF) the average signal within ROIs across time in the preprocessed fMRI data, combining the resultant neuronal timecourse with dummy-coded interaction regressors for each stimulus presentation, and then reconvolving the waveform with the HRF. The first-level models were similar to those above, except they also included a general timecourse for the ROI and separate timecourses merged with the dummy-coded contrast files for each stimulus condition (stimulation and attention/rest). We applied the same cluster extent and cluster forming threshold as above (i.e., *k* = 172, *p* = 0.005).

We next completed linear models to investigate associations between PCL-5 scores and BOLD reactivity in ROIs that exhibited significant effects. Average and differential responses were calculated and subject to separate models with the PCL-5 total scores. PCL-5 score outliers were evaluated using z-scores (>3SDs) but not excluded from analyses given the limited sample size. We investigated associations with PCL-5 subscale scores if significant associations with the total score were observed. A nominal *p*-value of *p* = 0.05 was used to determine significance. We finally completed an exploratory whole-brain analysis of the association between neural reactivity and PCL-5 scores within 3dMVM. The model included a within-subjects factor for stimulus and a between-subjects factor for PCL-5 scores at the MRI session, as well as interaction. We applied the same cluster extent and cluster forming threshold as above (i.e., *k* = 172, *p* = 0.005).

## Results

### Behavioral responses to the attention check

All NRTE participants had 100% accuracy (i.e., pressed the button during the red dot presentation) during the task (Fig. S2). A one-sample t-test within the RTE group revealed accuracy was significantly greater than zero [t(23) = 12.06, *p* < 0.001, M = 84%, SD = 34%], with four participants showing below 90% accuracy. An independent samples t-test on the ratio of button presses to number of trials (assessing multi-button presses during trials) did not reveal a significant difference between the NRTE and RTE groups [t(38) = 0.06, *p* = 0.954]. For further fMRI analyses, we set a minimum accuracy cut-off of 50% and excluded four participants in the RTE group. For completeness, we also completed sensitivity analyses using the button press ratios as a covariate in our initial voxel-wise linear mixed effects model (see Supplementary Material). Further, independent samples t-tests did not reveal differences between RTE and NRTE groups in reaction time regardless of focusing on individuals who did [t(34) = −1.15, *p* = 0.258] or did not [t(36) = −1.32, *p* = 0.196] pass our threshold for accuracy.

### Neural responses to visual stimulation

**Fig. 1:**
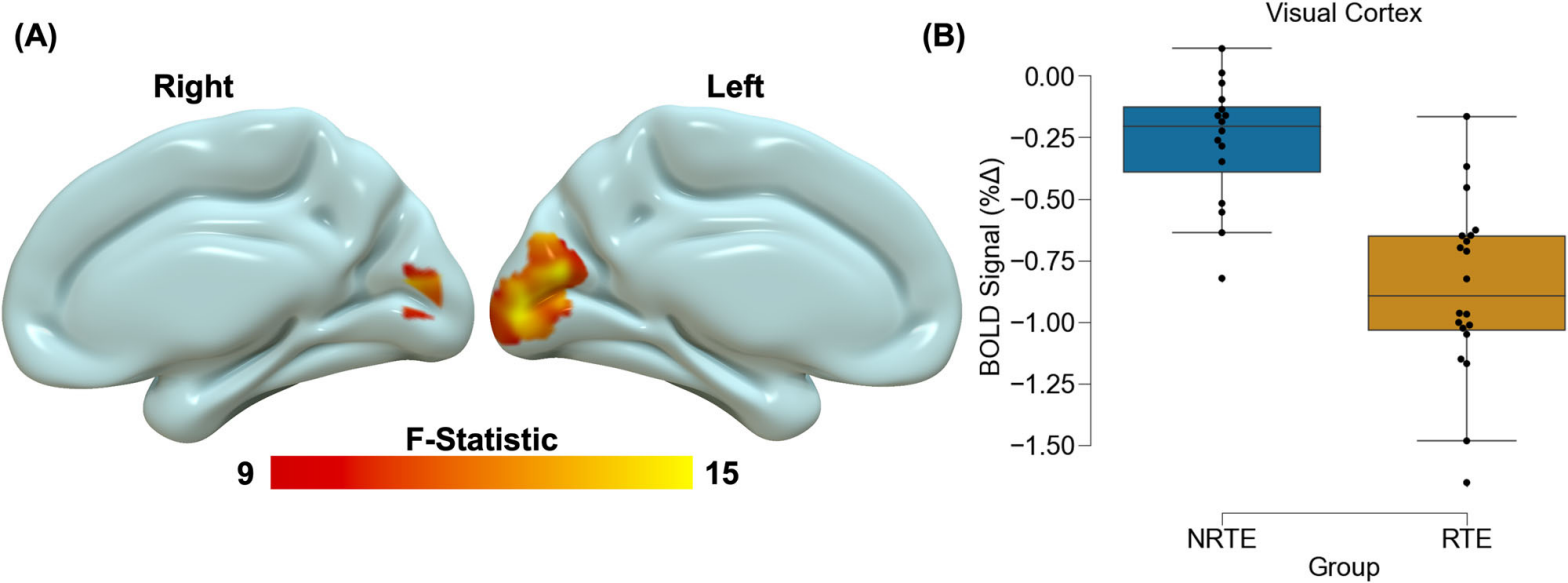
Trauma-related reductions in visual cortex reactivity. Recent trauma-exposed (RTE) individuals showed lower blood oxygen level-dependent (BOLD) signal responses compared to non-recent trauma-exposed (NRTE) individuals. The group-effect contrast revealed significant differences between RTE and NRTE individuals within the visual cortex **A**. Resultant signal extraction and descriptive plots of the data revealed the effect was reduced BOLD signal reactivity across all stimuli for the RTE group compared to the NRTE group **B**. Graph depicts the average BOLD signal across the cluster observed in **A**. The blue bar represents the NRTE group and the orange bar represents the RTE group. Black dots represent individual data points for each group. Inner bars within the boxplot represent the mean and outside bars represent the standard error.

**Fig. 2:**
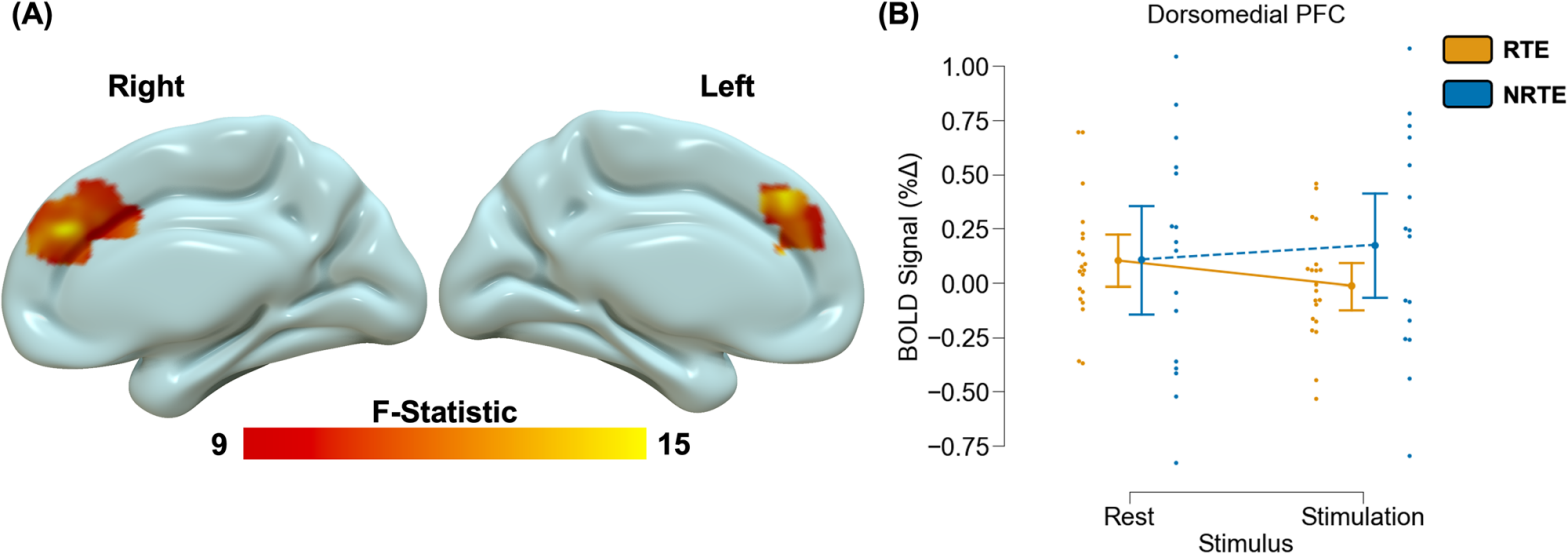
Trauma-related alterations in prefrontal processing during visual stimulation. A voxelwise multivariate model revealed a stimulus-by-group interaction effect within the dorsomedial PFC **A**. Specifically, recent trauma-exposed (RTE) individuals showed lower blood oxygen level-dependent (BOLD) signal responses to stimulation compared to attention/rest; however, non-recent trauma-exposed (NRTE) individuals showed greater BOLD signal responses to stimulation compared to attention/rest within this region **B**. Graph depicts the average BOLD signal across the cluster observed in **A**. Point plots represent the mean and 95% confidence interval for RTE (orange) and NRTE (blue) groups during attention/rest and stimulation. Dots represent individual data points for RTE (orange) and NRTE (blue) during attention/rest and stimulation.

Voxelwise analyses revealed significant main effects of stimulus type across the brain (Table 2; Fig. S3). Neural reactivity was greater during the stimulation than attention/rest condition within the clusters of the visual cortex, thalamus, and caudate. In contrast, neural reactivity was greater during the attention/rest than stimulation condition within prefrontal, parietal, and temporal/parahippocampal areas. Consistent with our hypothesis, we found a main effect of group within the visual cortex (Fig. 1) such that there was greater deactivation within the visual cortex for RTE participants compared to NRTE participants. Exploratory analyses of group differences of the individual conditions revealed greater deactivation in the RTE group during both stimulation and attention/rest (Table S4). The voxelwise analysis also revealed a significant group-by-stimulus-type interaction within the dorsomedial PFC (Fig. 2). Post-hoc analyses for the interaction effect revealed that RTE participants showed greater activation during attention/rest compared to stimulation within the dorsomedial PFC [t(19) = 5.98, *p* < 0.001_corrected_]. Conversely, NRTE participants showed greater activation during stimulation than attention/rest [t(15) = −2.96, *p* = 0.027_corrected_]. However, RTE and NRTE participants did not show significant between-group differences in dorsomedial PFC responses during attention/rest or stimulation (*p* > 0.05_corrected_).

**Table 2:** Voxelwise linear mixed effects model results

Region (Peak voxel)	Hemisphere	*F-*statistic	Volume (voxels)	Coordinates (MNI)		
Stimulus (Stimulation > Attention/Rest)				X	Y	Z
Primary visual cortex	Bilateral	314.06	15786	−4	−82	−4
Thalamus/Hippocampus^a^	Right	200.11	1605	24	−28	−2
Caudate	Right	25.59	200	16	24	14
Stimulus (Attention/Rest > Stimulation)						
SPL^a^	Right	99.73	6614	20	−58	68
Supramarginal gyrus	Left	42.4	3884	−58	−38	30
Middle temporal gyrus	Right	40.48	1825	50	−34	−6
Precuneus^a^	Right	32.88	1452	12	−72	30
	Left	99.97	308	−22	−50	12
	Right	49.1	184	26	−46	16
Superior frontal gyrus	Right	45.17	887	28	−10	62
Hippocampus/Parahippocampal gyrus	Left	27.86	397	−16	−22	−18
	Right	38.87	190	16	−22	−20
Paracentral gyrus	Left	34.23	241	−18	−14	64
Cerebellum	Right	31.05	238	8	−62	−18
Middle frontal gyrus	Right	19.92	200	24	54	20
Group						
Primary visual cortex	Bilateral	23.35	245	−4	−76	14
Interaction						
Dorsomedial PFC	Bilateral	26.4	206	4	40	30

F-statistic and coordinates are for the peak voxel in a cluster. Volume is given in *k*, the number of voxels (2 × 2 × 2) in the cluster.

*SPL* superior parietal lobule, *PFC* prefrontal cortex.

^a^Indicates cluster extends bilaterally.

### Neural connectivity during visual stimulation

**Fig. 3:**
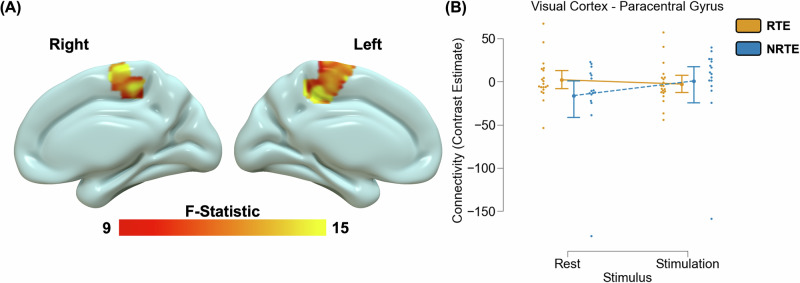
Trauma-related alterations in visual cortex to paracentral gyrus connectivity during visual stimulation. A voxelwise multivariate model revealed a stimulus-by-group interaction effect on paracentral gyrus to visual cortex connectivity **A**. Specifically, recent trauma-exposed (RTE) individuals showed no difference in functional connectivity between stimulation and attention/rest; however, non-recent trauma-exposed (NRTE) individuals showed greater connectivity during stimulation compared to attention/rest **B**. Graph depicts the average contrast estimate from the general psychophysiological (gPPI) model across the cluster observed in **A**. Point plots represent the mean and 95% confidence interval for RTE (orange) and NRTE (blue) groups during attention/rest and stimulation. Dots represent individual data points for RTE (orange) and NRTE (blue) during attention/rest and stimulation.

Voxelwise analyses did not reveal significant group differences in background connectivity using either the dmPFC or the visual cortex seed identified in our primary analyses. We did observe a significant main effect of stimulus (Table S3) and a group-by-stimulus-interaction on task-related connectivity between the visual cortex ROI and paracentral gyrus (Table S3; Fig. 3). Post hoc analyses revealed RTE participants did not show significant task-related differences in coupling between the visual cortex and paracentral gyrus [t(19) = 2.09, *p* > 0.05_corrected_]. In contrast, NRTE participants showed greater coupling during visual stimulation [t(15) = −6.46, *p* < 0.001_corrected_]. The pattern persisted even after removing one NRTE participant with extreme negative values [t(14) = −6.09, *p* < 0.001_corrceted_].

### PTSD symptoms and visual stimulation

Given our focus on visual processing in PTSD, we extracted the BOLD response from the visual cortex identified in the main effect of group and correlated both the differential (stimulation minus attention/rest) and the average BOLD response with PCL-5 scores within the RTE group. PCL-5 scores were negatively correlated with differential BOLD responses (stimulation minus attention/rest) within the RTE group [r(19) = −0.50, CI_95%_ = [−0.08, −0.77], *p* = 0.023] (Fig. S4). Follow-up analyses of symptom subscale scores revealed a significant association of differential BOLD response with intrusion [r(19) = −0.52, CI_95%_ = [−0.10, −0.78], *p* = 0.019], negative cognition and mood [r(19) = −0.50, CI_95%_ = [−0.08, −0.77], *p* = 0.024], and hyperarousal [r(19) = −0.48, CI_95%_ = [−0.01, −0.74], *p* = 0.048] scores, but not avoidance [r(19) = −0.33, CI_95%_ = [0.14, −0.67], *p* = 0.162] scores. We did not observe a significant interaction between group and the average BOLD response on PCL-5 scores (*p* > 0.05). The exploratory whole-brain analysis revealed a significant stimulus by PCL-5 interaction within the right inferior parietal lobule (Supplementary Material).

We completed additional analyses with PCL-5 scores collected 6 months after trauma exposure within the RTE group. Seven participants did not provide 6-month follow-up PCL-5 data. We did not observe significant associations with either the differential or average BOLD response with the visual cortex ROI within the RTE group (all *p* > 0.05). We then completed exploratory analyses using the significant interaction effect on neural reactivity (dmPFC ROI) and connectivity (paracentral gyrus ROI). We observed that 6-month PCL-5 scores were positively correlated with the average connectivity between the visual cortex and paracentral gyrus [r(13) = 0.77, CI_95%_ = [0.40, 0.92], *p* = 0.001] within the RTE group. The 6-month PCL-5 scores were not correlated with other contrasts (all *p* > 0.05).

## Discussion

Trauma exposure can lead to debilitating cognitive-affective dysfunction that is likely mediated by alterations in neurocircuitry involved in processing stimuli within the environment. While prior PTSD research has predominately focused on neural processing of affective information within threat neurocircuitry of trauma survivors, limited attention has been paid to processing of non-affective visual stimulation that may activate relevant circuitry for stimulus processing. In the present study, recent RTE and NRTE participants completed a visual stimulation task during fMRI. Compared to individuals without recent trauma exposure, recent trauma survivors exhibited greater deactivation within the primary visual cortex and altered neural reactivity to stimulation, compared to attention/rest, within the dorsomedial PFC. Further, connectivity between the visual cortex and paracentral gyrus during stimulation and attention/rest differed as a function of recent trauma exposure. Finally, neural reactivity within visual cortex was associated with PTSD symptoms within recent trauma survivors. The present findings suggest that trauma exposure may alter processing of basic visual information, potentiating downstream dysfunction in the form of PTSD symptoms.

Recent trauma exposure was associated with greater deactivation in the primary visual cortex. The visual cortex and its connected ventral visual stream form a primary pathway that supports the processing of both neutral and emotionally-valenced objects and other stimuli [26, 27]. Further, regions of the ventral visual stream play a role in memory and cognitive processes (e.g., vividness of mental imagery, object recognition) that may be related to PTSD-related dysfunction (for review, see [4]). There is limited prior work on neural reactivity to non-affective visual stimulation in recent trauma survivors. Previous research found that individuals with PTSD may show disruptions in visual object recognition. Dysfunctional recognition of affective stimuli is a core component of PTSD, with affected individuals often showing threat overgeneralization and impaired extinction recall [28–30]. Recent work also suggests that heightened PTSD symptoms are associated with reduced accuracy in visual object recognition and reduced relative power of evoked responses within prefrontal and temporal cortical areas [31]. Additionally, other research has shown that women with PTSD display increased visual cortex responses during self-referential processing, but decreased responses during evaluative judgments of others [32]. A key difference between the prior PTSD work and the present study is that, here, the trauma-related variability in visual responsivity occurred independently of stimulus valence (i.e., to neural stimulation). Further, in the present sample, PTSD symptoms were negatively related to neural reactivity to non-affective stimulation in recent trauma survivors. One possible interpretation is that recent trauma exposure induces changes in general processing of stimuli. Task-related deactivation (i.e., negative deflection from baseline) within the visual cortex across conditions for RTE individuals may suggest there is a general suppression of task-related neural reactivity in the acute phase following trauma. In contrast, the conversion to long-term PTSD may involve a shift towards more specific deficits in processing emotionally significant stimuli.

Recent trauma exposure additionally appeared to moderate both dorsomedial PFC reactivity and visual cortex to paracentral gyrus connectivity during stimulation compared to attention/rest. The dorsomedial PFC and its subcomponents are thought to play a role in detection of, and attention to, salient cues in the environment [33]. Further, both PTSD and trauma exposure are associated with disrupted dorsomedial PFC activity [34–36]. In a prior threat conditioning study [34], we observed that RTE individuals with lower expectations of experimental threat during presentations of safety showed greater dorsomedial PFC reactivity compared to those who did not. Taken with the present findings, one interpretation is that trauma exposure may induce a transient shift away from cognitive, effortful processing and towards more automatic processing of stimuli. For example, there may be a tendency toward dampened selective attention after trauma exposure, which could impede appropriate responses under task demands. RTE participants in the present study showed general deactivation of visual cortex during the task, as well as reduced dorsomedial PFC activation during stimulation. Trauma exposure may thus acutely reduce neural processing of stimuli in survivors. Further, NRTE participants showed modulation of visual to paracentral gyrus connectivity during stimulation that was not observed in RTE participants. The paracentral gyrus includes the motor cortex, which is important for initiating behavioral responses. Taken together, trauma exposure may lead to acute impairments in task engagement by disrupting threat-sensory-behavioral circuitry integration. In line with such reasoning, prior experimental work suggests that acute stress, with high task demands, can lead to shifts towards automatic processing in a prospective memory task [37]. Relatedly, our previously discussed experimental stress exposure has been associated with generalization of prior fear memories [38]. However, a caveat is that our prior work also suggests some RTE individuals may engage in compensatory neural processes to meet task demands, and the specific mechanisms underlying variability in such behavior remain unclear. Future research is needed to determine the acute effects of trauma exposure on cognitive-affective function.

The present findings should be considered along with several limitations. The sample consisted of individuals recruited from emergency departments, most of whom had experienced trauma from falls or motor vehicle accidents. Although other studies of trauma have utilized ED-based recruitment for recent trauma survivors [39, 40], our sample does not include other trauma types (e.g., sexual violence) that may impact affective visual circuitry [41, 42]. Additionally, the present design could have been improved by more objective measures of participant performance. While participants were instructed to fixate on a point on the screen, it is possible that RTE and NRTE participants differed in their eye movements in a manner that affected the results. Using eye-tracking measures alongside visual fMRI tasks in trauma survivors may help establish adherence to the task and provide novel insight into psychobiological impacts of trauma exposure. The limited sample size of the present study should also be considered in the interpretation of within-group brain and PTSD symptom associations. Smaller samples impose a restricted range of detectable correlations, which can bias such findings towards larger effect sizes. Additional research with larger samples is necessary to confirm the potential predictive utility of the observed brain imaging associations with PTSD symptoms. Larger samples would further allow for consideration of potential moderator variables – such as the timing of prior traumatic events– on the association between neural responses to visual stimulation and PTSD. In addition, future research could investigate potential sex differences or interactions with sex-related factors, given known associations of sex with PTSD. Finally, our study design was limited to simple visual stimulation to assess neural responsivity. Future research could use more advanced designs to selectively excite specific regions along the ventral visual stream, which may allow for more fine-grained analyses of circuit dysfunction related to trauma exposure.

In conclusion, recent trauma survivors showed differences in neural responsivity to basic visual stimulation compared to individuals without recent trauma exposure. Specifically, we observed altered reactivity within the visual cortex and dorsomedial PFC. Further, visual cortex responsivity in recent trauma survivors was associated with increased PTSD symptom severity. Taken together, our findings suggest that trauma exposure contributes to altered processing and responsivity of basic, non-affective visual information which – in turn – is related to PTSD symptom expression. The present findings provide novel insight into the neural underpinnings of PTSD.

## Supplementary information

Supplemental Methods
