# Supplementary material for: Traumatic stress alters neural reactivity to visual stimulation
Source: NPP Digit Psychiatry Neurosci. 2025 May 21;3:9. doi: 10.1038/s44277-025-00030-3 (PMC12095038; doi:10.1038/s44277-025-00030-3)
Supplement: Supplementary file 1 — Supplemental Methods [file 44277_2025_30_MOESM1_ESM.docx]

**Supplemental material to “*Traumatic stress alters neural reactivity to visual stimulation.”***

**Supplemental Methods**

**Anatomical MRI Data**

A T1-weighted multi-echo magnetization prepared rapid acquisition gradient echo (MEMPRAGE) structural scan was acquired (TR = 2500ms, TEs = 1.81/3.60/5.39/7.27ms, TI = 1000ms, flip angle = 8 degrees, FOV = 256mm, slices = 208, Voxel size = 0.8mm isotropic).

**FMRIPREP**

Below, we include the boilerplate methods from the fMRIPrep outputs for a single representative participant unchanged to maximize rigor and reproducibility of the present study:

Results included in this manuscript come from preprocessing performed using *fMRIPrep* 22.1.1 (Esteban, Markiewicz, et al. (2018); Esteban, Blair, et al. (2018); RRID:SCR_ 016216), which is based on *Nipype* 1.8.5 (K. Gorgolewski et al. (2011); K. J. Gorgolewski et al. (2018); RRID:SCR_002502).

**Preprocessing of B_0_ inhomogeneity mappings**

A total of 5 fieldmaps were found available within the input BIDS structure for this particular subject. A B_0_-nonuniformity map (or *fieldmap*) was estimated based on two (or more) echo-planar imaging (EPI) references with topup (Andersson, Skare, and Ashburner (2003); FSL 6.0.5.1:57b01774).

**Anatomical data preprocessing**

A total of 1 T1-weighted (T1w) images were found within the input BIDS dataset. The T1-weighted (Tw) image was corrected for intensity non-uniformity (INU) with N4BiasFieldCorrection (Tustison et al. 2010), distributed with ANTs 2.3.3 (Avants et al. 2008, RRID:SCR_004757), and used as T1w-reference throughout the workflow. The T1w-reference was then skull-stripped with a *Nipype* implementation of the antsBrainExtraction.sh workflow (from ANTs), using OASIS30ANTs as target template. Brain tissue segmentation of cerebrospinal fluid (CSF), white-matter (WM) and gray-matter (GM) was performed on the brain-extracted T1w using fast (FSL 6.0.5.1:57b01774, RRID:SCR_002823, Zhang, Brady, and Smith 2001). Brain surfaces were reconstructed using recon-all (FreeSurfer 7.2.0, RRID:SCR_001847, Dale, Fischl, and Sereno 1999), and the brain mask estimated previously was refined with a custom variation of the method to reconcile ANTs-derived and FreeSurfer-derived segmentations of the cortical gray-matter of Mindboggle (RRID:SCR_002438, Klein et al. 2017). Volume-based spatial normalization to two standard spaces (MNI152NLin6Asym, MNI152NLin2009cAsym) was performed through nonlinear registration with antsRegistration (ANTs 2.3.3), using brain-extracted versions of both T1w reference and the T1w template. The following templates were selected for spatial normalization: *FSL's MNI ICBM 152 non-linear 6th Generation Asymmetric Average Brain Stereotaxic Registration Model* [Evans et al.

(2012), RRID:SCR_002823; TemplateFlow ID: MNI152NLin6Asym], *ICBM 152 Nonlinear Asymmetrical template version 2009c* [Fonov et al. (2009), RRID:SCR_008796; TemplateFlow ID: MNI152NLin2009cAsym].

**Functional data preprocessing**

For each of the 5 BOLD runs found per subject (across all tasks and sessions), the following preprocessing was performed. First, a reference volume and its skull-stripped version were generated by aligning and averaging 4 single-band references (SBRefs). Head-motion parameters with respect to the BOLD reference (transformation matrices, and six corresponding rotation and translation parameters) are estimated before any spatiotemporal filtering using mcflirt (FSL 6.0.5.1:57b01774, Jenkinson et al. 2002). The estimated *fieldmap* was then aligned with rigid-registration to the target EPI (echo-planar imaging) reference run. The field coefficients were mapped on to the reference EPI using the transform. BOLD runs were slice-time corrected to 0.934s (0.5 of slice acquisition range Os-1.87s) using 3dTshift from AFNI (Cox and Hyde 1997, RRID:SCR_005927). A T2* map was estimated from the preprocessed EPI echoes, by voxel-wise fitting the maximal number of echoes with reliable signal in that voxel to a monoexponential signal decay model with nonlinear regression. The T2*/S_0_ estimates from a log-linear regression fit were used for initial values. The calculated T2* map was then used to optimally combine preprocessed BOLD across echoes following the method described in (Posse et al. 1999). The optimally combined time series was carried forward as the *preprocessed* BOLD. The BOLD reference was then co-registered to the T1w reference using bbregister (FreeSurfer) which implements boundary-based registration (Greve and Fischl 2009). Co-registration was configured with six degrees of freedom. First, a reference volume and its skull-stripped version were generated using a custom methodology of *fMRIPrep*. Several confounding time-series were calculated based on the *preprocessed* BOLD: framewise displacement (FD), DVARS and three region-wise global signals. FD was computed using two formulations following Power (absolute sum of relative motions, Power et al. (2014)) and Jenkinson (relative root mean square displacement between affines, Jenkinson et al. (2002). FD and DVARS are calculated for each functional run, both using their implementations in *Nipype* (following the definitions by Power et al. 2014). The three global signals are extracted within the CSF, the WM, and the whole-brain masks. Additionally, a set of physiological regressors were extracted to allow for component-based noise correction (*CompCor*, Behzadi et al. 2007). Principal components are estimated after high-pass filtering the preprocessed BOLD time-series (using a discrete cosine filter with 128s cut-off) for the two *CompCor* variants: temporal (tCompCor) and anatomical (aCompCor). tCompCor components are then calculated from the top 2% variable voxels within the brain mask. For aCompCor, three probabilistic masks (CSF, WM and combined CSF+WM) are generated in anatomical space. The implementation differs from that of Behzadi et al. in that instead of eroding the masks by 2 pixels on BOLD space, a mask of pixels that likely contain a volume fraction of GM is subtracted from the aCompCor masks. This mask is obtained by dilating a GM mask extracted from the FreeSurfer's *aseg* segmentation, and it ensures components are not extracted from voxels containing a minimal fraction of GM. Finally, these masks are resampled into BOLD space and binarized by thresholding at 0.99 (as in the original implementation). Components are also calculated separately within the WM and CSF masks. For each CompCor decomposition, the *k* components with the largest singular values are retained, such that the retained components' time series are sufficient to explain 50 percent of variance across the nuisance mask (CSF, WM, combined, or temporal). The remaining components are dropped from consideration. The head-motion estimates calculated in the correction step were also placed within the corresponding confounds file. The confound time series derived from head motion estimates and global signals were expanded with the inclusion of temporal derivatives and quadratic terms for each (Satterthwaite et al. 2013). Frames that exceeded a threshold of 0.5 mm FD or 1.5 standardized DVARS were annotated as motion outliers. Additional nuisance timeseries are calculated by means of principal components analysis of the signal found within a thin band (*crown*) of voxels around the edge of the brain, as proposed by (Patriat, Reynolds, and Birn 2017). The BOLD time-series were resampled into standard space, generating a *preprocessed BOLD run in MNI152NLin6Asym space*. First, a reference volume and its skull-stripped version were generated using a custom methodology of *fMRIPrep*. The BOLD time-series were resampled onto the following surfaces (FreeSurfer reconstruction nomenclature): *fsaverage*. Automatic removal of motion artifacts using independent component analysis (ICA-AROMA, Pruim et al. 2015) was performed on the *preprocessed BOLD on MNI space* time-series after removal of non-steady state volumes and spatial smoothing with an isotropic, Gaussian kernel of 6mm FWHM (full-width half-maximum). Corresponding "non-aggressively" denoised runs were produced after such smoothing. Additionally, the "aggressive" noise-regressors were collected and placed in the corresponding confounds file. All resamplings can be performed with *a single interpolation step* by composing all the pertinent transformations (i.e. head-motion transform matrices, susceptibility distortion correction when available, and co-registrations to anatomical and output spaces). Gridded (volumetric) resamplings were performed using antsApplyTransforms (ANTs), configured with Lanczos interpolation to minimize the smoothing effects of other kernels (Lanczos 1964). Non-gridded (surface) resamplings were performed using mri_vo12surf (FreeSurfer).

**Functional data preprocessing**

For each of the 5 BOLD runs found per subject (across all tasks and sessions), the following preprocessing was performed. First, a reference volume and its skull-stripped version were generated by aligning and averaging 4 single-band references (SBRefs). Head-motion parameters with respect to the BOLD reference (transformation matrices, and six corresponding rotation and translation parameters) are estimated before any spatiotemporal filtering using mcflirt (FSL 6.0.5.1:57b01774, Jenkinson et al. 2002). The estimated *fieldmap* was then aligned with rigid-registration to the target EPT (echo-planar imaging) reference run. The field coefficients were mapped on to the reference EPI using the transform. BOLD runs were slice-time corrected to 0.935s (0.5 of slice acquisition range Os-1.87s) using 3dTshift from AFNI (Cox and Hyde 1997, RRID:SCR_005927). A T2* map was estimated from the preprocessed EPI echoes, by voxel-wise fitting the maximal number of echoes with reliable signal in that voxel to a monoexponential signal decay model with nonlinear regression. The T2*/S_0_, estimates from a log-linear regression fit were used for initial values. The calculated T2* map was then used to optimally combine preprocessed BOLD across echoes following the method described in (Posse et al. 1999). The optimally combined time series was carried forward as the *preprocessed BOLD*. The BOLD reference was then co-registered to the T1w reference using bbregister (FreeSurfer) which implements boundary-based registration (Greve and Fischl 2009). Co-registration was configured with six degrees of freedom. First, a reference volume and its skull-stripped version were generated using a custom methodology of *fMRIPrep*. Several confounding time-series were calculated based on the *preprocessed BOLD*: framewise displacement (FD), DVARS and three region-wise global signals. FD was computed using two formulations following Power (absolute sum of relative motions, Power et al. (2014)) and Jenkinson relative root mean square displacement between affines, Jenkinson et al. (2002)). FD and DVARS are calculated for each functional run, both using their implementations in *Nipype* (following the definitions by Power et al. 2014). The three global signals are extracted within the CSF, the WM, and the whole-brain masks. Additionally, a set of physiological regressors were extracted to allow for component-based noise correction (*CompCor*, Behzadi et al. 2007). Principal components are estimated after high-pass filtering the *preprocessed BOLD time-series* (using a discrete cosine filter with 128s cut-off) for the two *CompCor* variants: temporal (tCompCor) and anatomical (aCompCor).

CompCor components are then calculated from the top 2% variable voxels within the brain mask. For aCompCor, three probabilistic masks (CSF, WM and combined CSF+WM) are generated in anatomical space. The implementation differs from that of Behzadi et al. in that instead of eroding the masks by 2 pixels on BOLD space, a mask of pixels that likely contain a volume fraction of GM is subtracted from the aCompCor masks. This mask is obtained by dilating a GM mask extracted from the FreeSurfer's *aseg* segmentation, and it ensures components are not extracted from voxels containing a minimal fraction of GM. Finally, these masks are resampled into BOLD space and binarized by thresholding at 0.99 (as in the original implementation). Components are also calculated separately within the WM and CSF masks. For each CompCor decomposition, the *k* components with the largest singular values are retained, such that the retained components' time series are sufficient to explain 50 percent of variance across the nuisance mask (CSF, WM, combined, or temporal). The remaining components are dropped from consideration. The head-motion estimates calculated in the correction step were also placed within the corresponding confounds file. The confound time series derived from head motion estimates and global signals were expanded with the inclusion of temporal derivatives and quadratic terms for each (Satterthwaite et al. 2013). Frames that exceeded a threshold of 0.5 mm FD or 1.5 standardized DVARS were annotated as motion outliers. Additional nuisance timeseries are calculated by means of principal components analysis of the signal found within a thin band (*crown*) of voxels around the edge of the brain, as proposed by (Patriat, Reynolds, and Birn 2017). The BOLD time-series were resampled into standard space, generating a preprocessed BOLD run in MNI152NLin6Asym space. First, a reference volume and its skull-stripped version were generated using a custom methodology of *fMRIPrep*. The BOLD time-series were resampled onto the following surfaces (FreeSurfer reconstruction nomenclature): *fsaverage*. Automatic removal of motion artifacts using independent component analysis (ICA-AROMA, Pruim et al. 2015) was performed on the *preprocessed BOLD on MNI space* time-series after removal of non-steady state volumes and spatial smoothing with an isotropic, Gaussian kernel of 6mm FWHM (full-width half-maximum). Corresponding "non-aggressively" denoised runs were produced after such smoothing. Additionally, the "aggressive" noise-regressors were collected and placed in the corresponding confounds file. All resamplings can be performed with *a single interpolation step* by composing all the pertinent transformations (i.e. head-motion transform matrices, susceptibility distortion correction when available, and co-registrations to anatomical and output spaces). Gridded (volumetric) resamplings were performed using antsApplyTransforms (ANTs), configured with Lanezos interpolation to minimize the smoothing effects of other kernels (Lanczos 1964). Non-gridded (surface) resamplings were performed using mri_vol2surf (FreeSurfer).

Many internal operations of *fMRIPrep* use *Nilearn* 0.9.1 (Abraham et al. 2014, RRID:SCR_001362), mostly within the functional processing workflow. For more details of the pipeline, see the section corresponding to workflows in *fMRIPrep's* documentation.

**Copyright Waiver**

The above boilerplate text was automatically generated by fMRIPrep with the express intention that users should copy and paste this text into their manuscripts *unchanged*. It is released under the CCO license.

**Additional multi-echo fMRI Processing**

For multi-echo fMRI processing, we calculated a T2* map across echo times to create a single, optimally combined time series. The first echo of the fMRI task’s corresponding fieldmap was used for susceptibility distortion correction and applied to the combined time series.

**Sensitivity analyses for primary MRI models**

We completed sensitivity analyses for our primary linear-mixed effects models to investigate the effects of trauma exposure and stimulus type on neural reactivity (BOLD signal response), given the apparent variability in the ratio of button presses during the attention check for the RTE group. Sensitivity analyses were completed in two approaches. First, we completed factorial ANCOVAs with a between-subject factor of trauma exposure, a within-subjects factor of stimulus type, and a covariate of button press ratios for each participant on extracted BOLD signal responses for both a) the main effect of group within visual cortex and b) the interaction effect within the dorsomedial PFC. Second, we completed an additional linear mixed-effects model as described in the main methods, including a covariate for button press ratios.

**Supplementary Results**

**Normality tests of psychometric assessments**

PCL-5 and PROMIS depression scores were non-normally distributed for the RTE and NRTE groups (p < 0.05). LEC-5 scores (both current and past trauma) were normally distributed for both groups (p > 0.05). CTQ scores were non-normally distributed for both groups (p > 0.05).

**Sensitivity analyses for primary MRI models**

With the inclusion of button press ratios as a covariate using extracted BOLD signal responses from the mixed model findings, our factorial ANCOVA still showed both a significant effect of group on BOLD signal responses in the visual cortex [F(1,33) = 6.99, p < 0.001] and a significant interaction between group and stimulus type on BOLD signal responses within the dorsomedial PFC [F(1,33) = 29.75, p < 0.001]. Post-hoc tests for the interaction revealed a significant difference between BOLD signal responses to the rest and stimulation conditions for the RTE group [t(19) = 5.45, p < 0.05_corrected_], but not the NRTE group [t(15) = -2.68, p > 0.05_corrected_].

Voxelwise sensitivity analyses using button press ratios as a covariate revealed a main effect of group within the visual cortex [F-statistic_Peak_ = 27.52, *k* = 299, (XYZ_Peak_ = -2, -80, 8)] similar to the effect observed in the main analysis. We also observed an interaction effect within the dorsomedial PFC that fell below our cluster extent threshold [F-Statistic_Peak_ = 21.13, *k* = 109, (XYZ_Peak_ = 4, 40, 30)]. Taken together, the sensitivity analyses suggest our findings are largely robust to adjustments for behavioral responses. The changes in dorsomedial PFC cluster size may reflect altered task engagement in line with our speculative interpretation in the main manuscript.

**Exploratory whole-brain analysis with PCL-5 scores**

The exploratory whole-brain analysis revealed a significant stimulus by PCL-5 interaction within the right inferior parietal lobule for the RTE group [F_Peak_ = 44.86, p < 0.05_corrected_, XYZ_Peak_ = (50, -58, 24)]. Post-hoc analyses on the signal extracted from the cluster revealed associations with PCL-5 scores were positive for the stimulation condition [r = 0.17, p = 0.486], but negative for the rest condition [r = -0.37, p = 0.112].

**Supplementary References**

Abraham, Alexandre, Fabian Pedregosa, Michael Eickenberg, Philippe Gervais, Andreas Mueller, Jean Kossaifi, Alexandre Gramfort, Bertrand Thirion, and Gael Varoquaux. 2014. "Machine Learning for Neuroimaging with Scikit-Learn." *Frontiers in Neuroinformatics*

8. https://doi.org/10.3389/fninf.2014.00014.

Andersson, Jesper L. R., Stefan Skare, and John Ashburner. 2003. "How to Correct Susceptibility Distortions in Spin-Echo Echo-Planar Images: Application to Diffusion Tensor Imaging." *Neurolmage* 20 (2): 870-88. https://doi.org/10.1016/S1053-8119(03)00336-7.

Avants, B. B., C. L. Epstein, M. Grossman, and J. C. Gee. 2008. "Symmetric Diffeomorphic Image Registration with Cross-Correlation: Evaluating Automated Labeling of Elderly and Neurodegenerative Brain." *Medical Image Analysis* 12 (1): 26-41. https://doi.org/10.1016/j.media.2007.06.004.

Behzadi, Yashar, Khaled Restom, Joy Liau, and Thomas T. Liu. 2007. "A Component Based Noise Correction Method (CompCor) for BOLD and Perfusion Based fMRI." *Neurolmage* 37 (1): 90-101. https://doi.org/10.1016/j.neuroimage.2007.04.042.

Cox, Robert W., and James S. Hyde. 1997. "Software Tools for Analysis and Visualization of fMRI Data." *NMR in Biomedicine* 10 (4-5): 171-78. https://doi.org/10.1002/(SICI) 1099-1492(199706/08)10:4/5<171::AID-NBM453 >3.0.CO;2-L.

Dale, Anders M., Bruce Fischl, and Martin I. Sereno. 1999. "Cortical Surface-Based Analysis: I. Segmentation and Surface Reconstruction." *Neurolmage* 9 (2): 179-94. https://doi.org/10.1006/nimg.1998.0395.

Esteban, Oscar, Ross Blair, Christopher J. Markiewiez, Shoshana L. Berleant, Craig Moodie, Feilong Ma, Ayse Ilkay Isik, et al. 2018.

"fMRIPrep 22.1.1." *Software*. https://doi.org/10.5281/zenodo.852659.

Esteban, Oscar, Christopher Markiewicz, Ross W Blair, Craig Moodie, Ayse Ilkay Isik, Asier Erramuzpe Aliaga, James Kent, et al. 2018. "fMRIPrep: A Robust Preprocessing Pipeline for Functional MRI." Nature Methods. https://doi.org/10.1038/s41592-018-0235-4.

Evans, AC, AL Janke, DL Collins, and S Baillet. 2012. "Brain Templates and Atlases." *Neurolmage* 62 (2): 911-22. https://doi.org /10.1016/j.neuroimage.2012.01.024.

Fonov, VS, AC Evans, RC McKinstry, CR Almli, and DL Collins. 2009. "Unbiased Nonlinear Average Age-Appropriate Brain Templates from Birth to Adulthood." *Neurolmage* 47, Supplement 1: S102. https://doi.org/10.1016/S1053-8119(09)70884-5.

Gorgolewski, K., C. D. Burns, C. Madison, D. Clark, Y. O. Halchenko, M. L. Waskom, and S. Ghosh. 2011. "Nipype: A Flexible, Lightweight and Extensible Neuroimaging Data Processing Framework in Python." *Frontiers in Neuroinformatics* 5: 13. https://doi.org /10.3389/fninf.2011.00013.

Gorgolewski, Krzysztof J., Oscar Esteban, Christopher J. Markiewiez, Erik Ziegler, David Gage Ellis, Michael Philipp Notter, Dorota Jarecka, et al. 2018. "Nipype." *Software*. https://doi.org/10.5281/zenodo.596855.

Greve, Douglas N, and Bruce Fischl. 2009. "Accurate and Robust Brain Image Alignment Using Boundary-Based Registration." *Neurolmage* 48 (1): 63-72. https://doi.org/10.1016/j.neuroimage.2009.06.060.

Jenkinson, Mark, Peter Bannister, Michael Brady, and Stephen Smith. 2002. "Improved Optimization for the Robust and Accurate Linear Registration and Motion Correction of Brain Images." *Neurolmage* 17 (2): 825-41. https://doi.org/10.1006/nimg.2002.1132.

Klein, Arno, Satrajit S. Ghosh, Forrest S. Bao, Joachim Giard, Yrjö Häme, Eliezer Stavsky, Noah Lee, et al. 2017. "Mindboggling Morphometry of Human Brains." *PLOS Computational Biology* 13 (2): e1005350. https://doi.org/10.1371/journal.pcbi.1005350.

Lanczos, C. 1964. "Evaluation of Noisy Data." *Journal of the Society for Industrial and Applied Mathematics Series B Numerical Analysis* 1(1): 76-85. https://doi.org/10.1137/0701007.

Patriat, Rémi, Richard C. Reynolds, and Rasmus M. Birn. 2017. "An Improved Model of Motion-Related Signal Changes in fMRI." *Neurolmage* 144, Part A (January): 74-82. https://doi.org/10.1016/j.neuroimage.2016.08.051.

Posse, Stefan, Stefan Wiese, Daniel Gembris, Klaus Mathiak, Christoph Kessler, Maria-Lisa Grosse-Ruyken, Barbara Elghahwagi, Todd Richards, Stephen R. Dager, and Valerij G. Kiselev. 1999. "Enhancement of BOLD-Contrast Sensitivity by Single-Shot Multi-Echo Functional MR Imaging." *Magnetic Resonance in Medicine* 42 (1): 87-97. https://doi.org/10.1002

/(SICI) 1522-2594(199907) 42:1 <87::AID-MRM13>3.0.CO;2-0.

Power, Jonathan D., Anish Mitra, Timothy O. Laumann, Abraham Z. Snyder, Bradley L. Schlaggar, and Steven E. Petersen. 2014. "Methods to Detect, Characterize, and Remove Motion Artifact in Resting State MRI." *Neurolmage* 84 (Supplement C): 320-41.

https://doi.org/10.1016/j.neuroimage.2013.08.048.

Pruim, Raimon H. R., Maarten Mennes, Daan van Rooij, Alberto Llera, Jan K. Buitelaar, and Christian F. Beckmann. 2015. "ICA-AROMA: A Robust ICA-Based Strategy for Removing Motion Artifacts from MRI Data." *Neurolmage* 112 (Supplement C): 267-77. https://doi.org/10.1016/j.neuroimage.2015.02.064.

Satterthwaite, Theodore D., Mark A. Elliott, Raphael T. Gerraty, Kosha Ruparel, James Loughead, Monica E. Calkins, Simon B. Eickhoff, et al. 2013. "An improved framework for confound regression and filtering for control of motion artifact in the preprocessing of resting-state functional connectivity data." *Neurolmage* 64 (1): 240-56. https://doi.org/10.1016

/j.neuroimage.2012.08.052.

Tustison, N. J., B. B. Avants, P. A. Cook, Y. Zheng, A. Egan, P. A. Yushkevich, and J. C. Gee. 2010. "N4itk: Improved N3 Bias Correction." *IEEE Transactions on Medical Imaging* 29 (6): 1310-20. https://doi.org/10.1109/TMI.2010.2046908.

Zhang, Y., M. Brady, and S. Smith. 2001. "Segmentation of Brain MR Images Through a Hidden Markov Random Field Model and the Expectation-Maximization Algorithm." *IEEE Transactions on Medical Imaging* 20 (1): 45-57. https://doi.org/10.1109/42.906424.

**Table S1. Broad class trauma exposures for recent trauma survivors**

| **Trauma Type** | **Frequency** |
| --- | --- |
| Motor Vehicle Collision | 15 |
| Other  (Fall, Animal Injury, Assault) | 9 |

**Table S2. Traumatic events endorsed on the LEC-5**

| LEC-5 Event Type | **RTE** | | **NRTE** | |
| --- | --- | --- | --- | --- |
|  | Count | Percentage | Count | Percentage |
| >0 All trauma | 24 | 100.00% | 13 | 81.25% |
| >0 Past Trauma | 24 | 100.00% | - | - |
| Natural Disaster | 19 | 79.17% | 9 | 56.25% |
| Fire | 18 | 75.00% | 6 | 37.50% |
| Transport Accident | 24 | 100.00% | 11 | 68.75% |
| Serious Accident | 19 | 79.17% | 6 | 37.50% |
| Toxic Substance | 8 | 33.33% | 6 | 37.50% |
| Physical Assault | 19 | 79.17% | 8 | 50.00% |
| Assault with Weapon | 15 | 62.50% | 4 | 25.00% |
| Sexual Assault | 15 | 62.50% | 5 | 31.25% |
| Other Sexual Experience | 16 | 66.67% | 7 | 43.75% |
| War Exposure | 10 | 41.67% | 4 | 25.00% |
| Captivity | 6 | 25.00% | 2 | 12.50% |
| Life-Threatening Illness | 15 | 62.50% | 8 | 50.00% |
| Human Suffering | 15 | 62.50% | 6 | 37.50% |
| Violent Death | 17 | 70.83% | 4 | 25.00% |
| Accidental Death | 14 | 58.33% | 7 | 43.75% |
| Harm to Other | 4 | 16.67% | 1 | 6.25% |
| Other | 13 | 54.17% | 6 | 37.50% |

**Note:** >0 Past trauma not provided for NRTE group as value is same as >0 All Trauma. LEC-5: Life Events Checklist for DSM-5; TE: Trauma-Exposed; NRTE: Non-Recent Trauma-Exposed

**Table S3.**

| **Region (Peak voxel)** | **Hemisphere** | ***F-*statistic** | **Volume (voxels)** | **Coordinates (MNI)** | | |
| --- | --- | --- | --- | --- | --- | --- |
| *Stimulus* |  |  |  | X | Y | Z |
| Postcentral gyrus | Left | 37.55 | 1053 | -34 | -36 | 60 |
|  | Right | 30.69 | 269 | 54 | -30 | 36 |
| Paracentral lobule | Left | 44.02 | 640 | -10 | -32 | 50 |
| Precentral gyrus | Left | 34.18 | 441 | -36 | -24 | 54 |
|  | Right | 23.9 | 330 | 22 | -20 | 72 |
| Inferior parietal lobule | Right | 30.16 | 436 | 38 | -40 | 50 |
| Superior Temporal Gyrus | Right | 27.61 | 406 | 56 | -36 | 20 |
| Superior Parietal lobule | Left | 28.39 | 400 | -10 | -80 | 50 |
| Putamen | Left | 28.17 | 384 | -26 | 0 | -8 |
|  | Right | 28.04 | 276 | 28 | -20 | 14 |
| Cerebellum | Left | 38.16 | 346 | -28 | -44 | -22 |
|  | Right | 36.43 | 306 | 22 | -60 | -16 |
| Superior frontal gyrus | Right | 26.77 | 218 | 26 | 2 | 64 |
| *Interaction* |  |  |  |  |  |  |
| Paracentral gyrus* | Left | 28.18 | 294 | -10 | -34 | 50 |

Note: *Indicates cluster extends bilaterally. F-statistic and coordinates are for the peak voxel in a cluster. Volume is given in *k,* the number of voxels (2x2x2) in the cluster.

**Table S4.**

|  |  | **RTE** | **NRTE** |
| --- | --- | --- | --- |
| **Condition** | **T-statistic (p-value)** | **Mean (SD)** | **Mean (SD)** |
| Stimulation | 3.02 (<0.001) | -0.21 (0.28) | 0.04 (0.21) |
| Rest | 4.77 (0.005) | -1.52 (0.65) | -0.57 (0.49) |

**Figure S1. Schematic overview of the visual stimulation task.** Participants were asked to fixate on a yellow cross at the center of the screen during presentation of a full-field flickering checkerboard (8Hz). The checkerboard was presented for 15 seconds. Participants were asked to press a button on an MRI-compatible button box when a red dot was presented, which served as a rest condition for another 15 seconds. Blocks of checkerboard and rest were presented 14 times per participant.

**
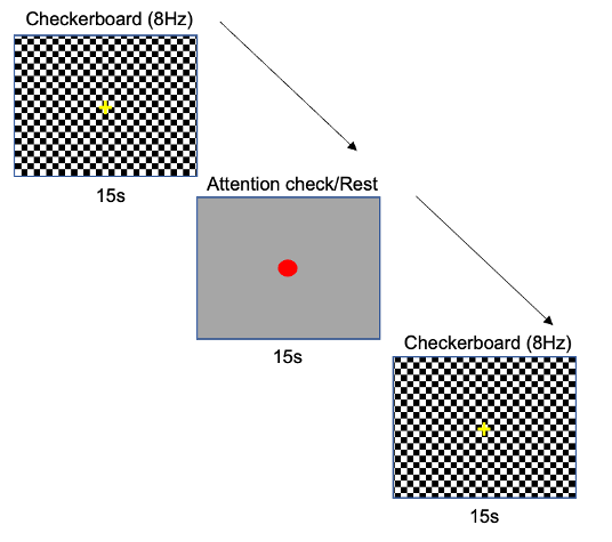
**

**Figure S2. Trauma-related variability in behavior during visual stimulation.** Independent t-tests were completed to assess potential group differences in accuracy (i.e., participants pressed button at least once during presentation of red-dot; A) and the ratio of button presses (i.e., the number of button presses during red-dot trials/the number of red dot trials; B). Groups did not differ in accuracy or button press ratios, though several TE participants did not show high accuracy. Graph depicts the values for RTE and NRTE groups. The blue bar represents the NRTE group and the orange bar represents the RTE group. Black dots represent individual data points for each group. Inner bars within the boxplot represent the mean and outside bars represent the standard error.


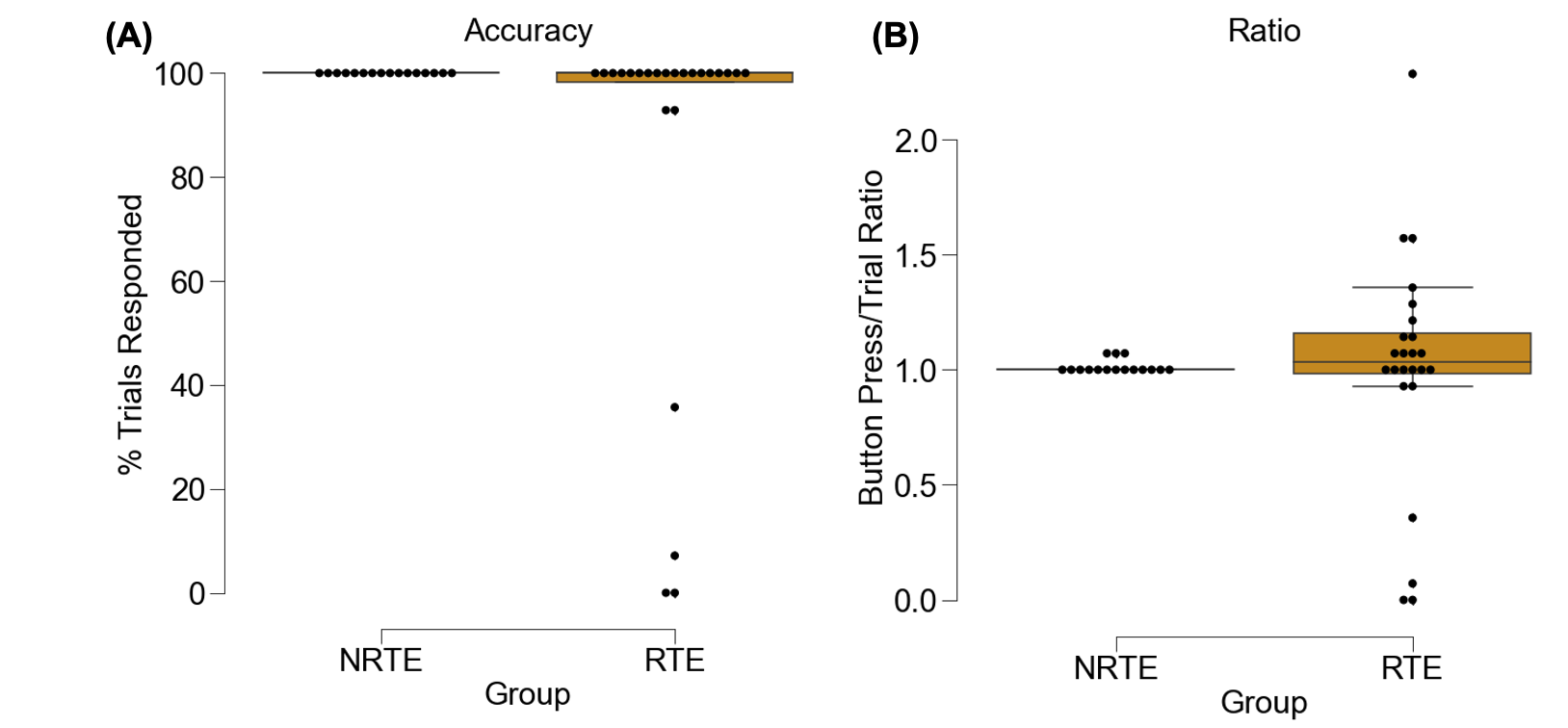


**Figure S3. Main effect of stimulus across participants.** A voxelwise analysis revealed a significant main effect of stimulus across several regions including the visual cortex, superior parietal lobule, and prefrontal cortex.

**
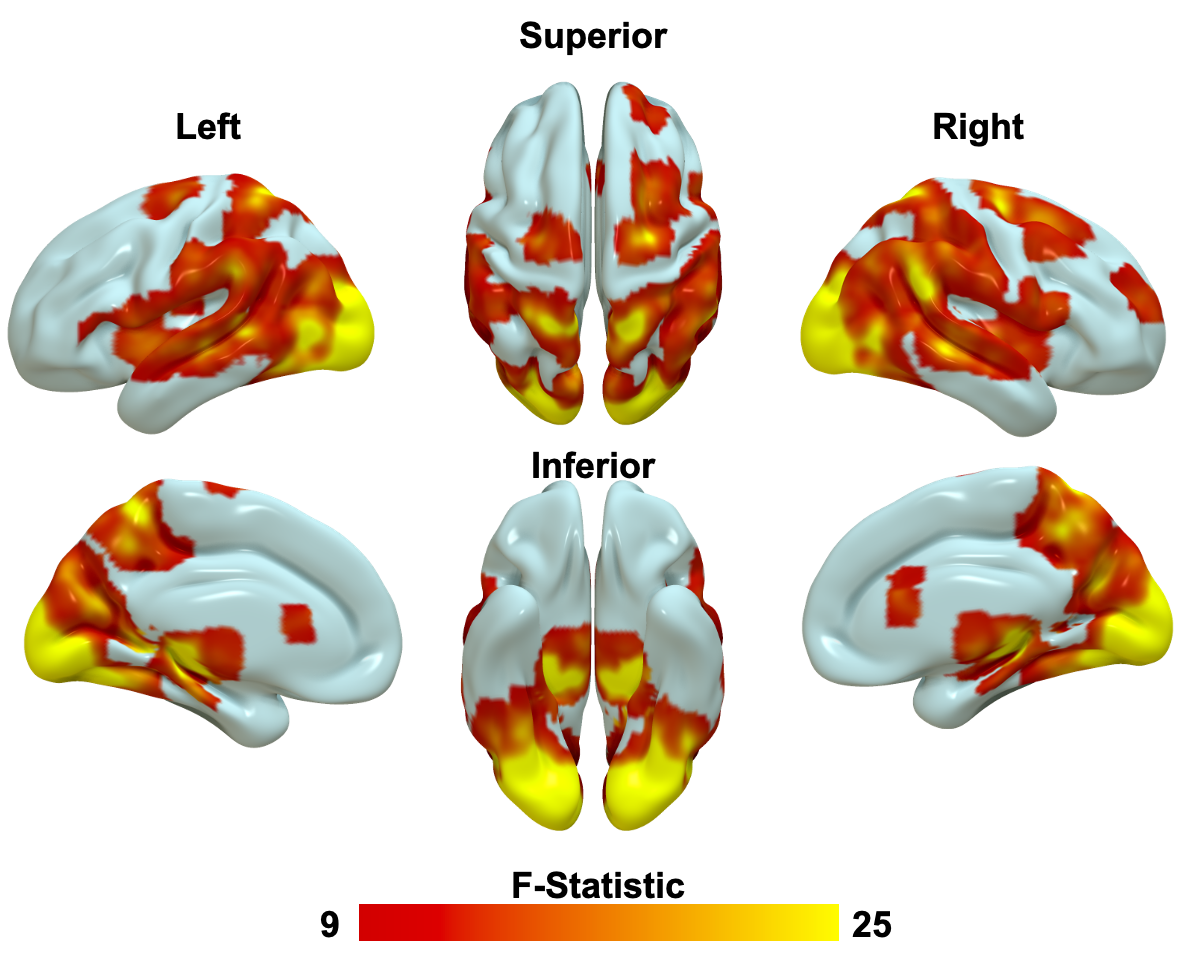
**

**Figure S4. PTSD symptoms vary with differential neural reactivity within the visual cortex.** A correlation analysis revealed that, within the TE group, differential (Stimulation – Rest) reactivity within the visual cortex was negatively associated with PTSD symptoms indexed via the PTSD Checklist for DSM-5 (PCL-5). Graph depicts the linear association between PCL-5 total scores and differential BOLD signal responses for each participant. Points represent individual data points, the solid line represents the linear line of best fit, and the shaded regions represent the 95% confidence interval.

**
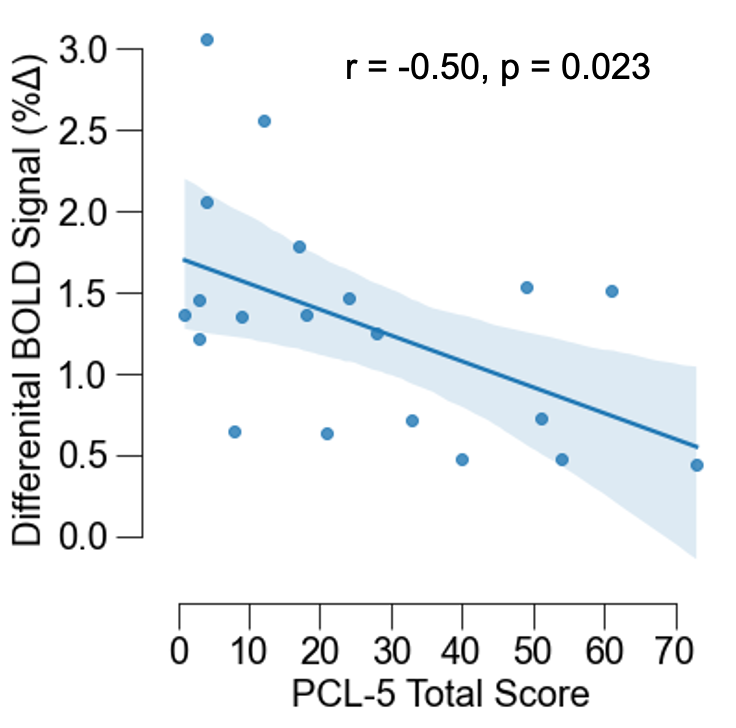
**
